# Quantitative Analysis of Cereulide Toxin from *Bacillus cereus* in Rice and Pasta Using Synthetic Cereulide Standard and ^13^C_6_-Cereulide Standard—A Short Validation Study

**DOI:** 10.3390/toxins6123326

**Published:** 2014-12-11

**Authors:** Aida Zuberovic Muratovic, Rikard Tröger, Kristina Granelli, Karl-Erik Hellenäs

**Affiliations:** Science Department, National Food Agency, Box 622, SE-751 26 Uppsala, Sweden; E-Mails: rikard.troger@outlook.com (R.T.); kristina.granelli@slv.se (K.G.); karl-erik.hellenas@slv.se (K.-E.H.)

**Keywords:** cereulide, toxin, *Bacillus cereus*, validation, foods, UPLC-MS/MS

## Abstract

A single laboratory validation study of a rapid and sensitive quantitative method for the analysis of cereulide toxin produced by *Bacillus cereus* using ultra high performance liquid chromatography-electrospray-tandem mass spectrometry is presented. The analysis of this cyclic peptide toxin was validated for pasta and rice samples using a newly presented synthetic cereulide peptide standard, together with ^13^C_6_-cereulide that previously have not been commercially available. The use of cereulide standard was also compared to the most frequently used surrogate standard, the antibiotic valinomycin. The performance of the method was evaluated by analyzing spiked sample pools from different types of rice and pasta, as well as 21 individual rice and pasta samples from differently prepared meals. Inoculation of samples with three cereulide toxin-producing strains of *Bacillus cereus* was finally used to mimic naturally contaminated foods. The quantification range of the method was 1–500 ng/g (*R*^2^ = 0.999) and the limits of detection and quantification were 0.1 and 1 ng/g, respectively. The precision varied from 3% to 7% relative standard deviation and the trueness from −2% to +6% relative bias at different concentration levels in cooked rice and pasta.

## 1. Introduction

Cereulide toxine produced by *Bacillus cereus*, a Gram-positive bacteria, is a common cause of food poisoning [1,2]. The symptoms are characterized by nausea and vomiting that can be accompanied by diarrheal syndrome in approximately a third of the cases [3]. The intoxication may be severe and can lead to fatal outcomes [4,5,6]. Although, the diarrheal part of the illness is caused by enterotoxins produced in the small intestine after ingestion of bacterial cells/spores, and not by cereulide, the combination of symptoms pronounces the seriousness of the illness [7]. The production of toxins has been observed as a bacterial response to the environmental conditions like pH, temperature, oxygen tension and high starch content in foods like rice and pasta dishes [2,8]. The cereulide toxin is as a cyclic dodecadepsipeptide with a molecular mass of 1.2 kDa and exceptional stability properties which make it difficult to inactivate in foods [9,10,11]. These properties include the resistance to digestion enzymes like pepsin and trypsin, extreme pH conditions and even heat at temperatures of up to 150 °C [12]. The toxin is pre-formed in foods and is able to retain its biological toxic activity throughout the human acidic stomach environment and the digestion region.

The human toxic dose of cereulide has not been determined, but has been estimated from animal studies or *in vitro* experiments to be ~8 μg/kg body weight [9,13,14,15]. The lowest illness-inducing doses of cereulide toxin in foods that have been reported to now are at low nanogram level, ~5–10 ng of cereulide/g of foods [16,17]. At lower doses, cereulide might give rise to symptoms of milder nature or diffuse enough that might pass unrecorded.

Several types of detection methods for cereulide have been presented, among which a bioassay based on boar spermatozoa motility is reported to be one of the most easily performed [14,18]. Other methods include cytotoxicity based assays in cell cultures like rat liver cells [19], HEP-2 cells [20] or CHO (Chinese hamster ovary) cells [21]. Along with the difficulties to produce immunoassay based methods for cereulide toxin [16], the bioassay based methods are known to suffer from interferences with molecules that have similar properties as the analyte of interest causing cross-reactivity and consequently luck of specificity for the analysis. Using chemical methods most of such issues can be circumvented. Several reports on successful quantitative analysis of cereulide using liquid chromatography coupled to mass or tandem mass spectrometry (LC-MS and MS/MS) have been presented [16,22,23,24]. However, as a synthetic cereulide standard has not been commercially available a surrogate standard, like the antibiotic valinomycin, had to be used as the best choice in previous studies where the content of cereulide in food samples was expressed in valinomycin equivalents. Recently, the synthetic cereulide standard was developed that has become commercially available and used in this study [25].

In this report, we present a short in-house validation of a method using the novel synthetic cereulide standard together with ^13^C_6_-labeled cereulide as internal standard for quantitative analysis of cereulide toxin in rice and pasta samples with UPLC-ESI-MS/MS. This is, to the best of our knowledge, the first report on the quantitative MS analysis of cereulide using original standard. The usage of original standard eliminates possible ionization differences, due to matrix effects interferences in the ES-ionization step (like signal enhancement or suppression) [26] that might exist when using surrogate standards. This, in combination with the benefits of the fragmentation in tandem mass spectrometry provides maximal specificity and increases the robustness and accuracy of the analysis. The presented method is easily performed and sensitive which together with its rapidness and specificity makes it be the best choice in emergent scenarios when it is important to distinguish the food-born disease caused by low levels of cereulide from *B. cereus* from other food intoxications that give similar symptoms, in order to protect human health.

## 2. Results and Discussion

### 2.1. Validation Design

To set-up and validate the UPLC-ESI-MS/MS method for quantitative analysis of cereulide toxin cooked rice and pasta samples of different types were prepared, pooled and spiked with cereulide toxin standard. For the quantitative analysis of cereulide a newly presented commercially available synthetic cereulide standard and ^13^C_6_-internal standard of cereulide were used [25].

### 2.2. Calibration and Linearity

**Figure 1:**
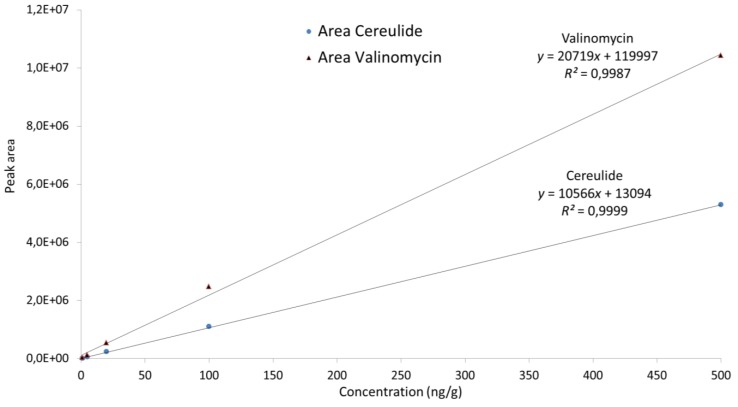
Comparison of calibration curves for cereulide standard and valinomycin. Area is plotted as a function of the concentration for each of the compounds.

**Figure 2:**
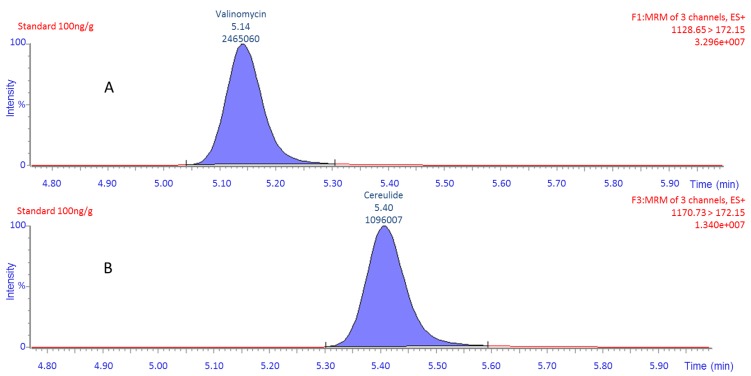
MRM spectrum (multiple reaction monitoring-MRM) of the same product ions (*m*/*z* 172.15) for cereulide standard (**B**) and valinomycin (**A**) at a concentration of 100 ng/g. The peak annotations show the retention time and area.

Linearity and possible matrix effects during the UPLC-MS/MS analysis were evaluated by comparing calibration curves prepared from the analysis of extracts of the pooled samples of each matrix and the methanol/water mixtures both spiked with cereulide at 0, 1, 5, 20, 100 and 500 ng/g. The derived calibration curves were linear in the tested concentration range (*R*^2^ were for solvent/water: 1.000, pasta extract: 0.9999 and rice extract: 0.9998) and no evidence of matrix effects could be identified. The calibration curve of the cereulide standard was compared to the corresponding calibration curve of valinomycin (Figure 1). The diagram visualizes the difference in signal response between these two compounds, which, under the analytical conditions applied in the present method, would result in an underestimation of the cereulide content by a factor of two. Furthermore, as Figure 2 shows, the differences between cereulide and valinomycin are visible also in chromatography where these two compounds were compared at the same concentration and for the same product ion (*m*/*z* 172.15). This confirms the advantages of using the cereulide standard compared to valinomycin.

### 2.3. Precision, Trueness and Recovery

The precision and trueness of the method were studied by quantitative analysis of pooled rice and pasta samples spiked at three different concentrations (1, 10 and 100 ng/g). The results were similar for both food types, with relative standard deviation (RSD) values ranging from 3% to 7% and bias values from −2% to +6% (Table 1). In another experiment, the possible influence on sample matrix variations on precision and trueness was investigated by spiking 21 different authentic food samples of rice and pasta, respectively. The RSD and bias values were within the ranges obtained for pooled samples, indicating that the method is robust towards sample matrix effects. The absolute extraction recovery, obtained from comparative analysis of samples spiked before and after extraction, was determined to be 91%–93%, not differing significantly between pasta and rice. The extraction losses will not influence the quantitative results of the method, as estimated by the trueness figures given above, since the internal standard used for quantification is added prior to extraction. The results from the experiments on precision, trueness and recovery are summarized in Table 1.

**Table 1:** Results from experiments on trueness, precision and extraction recovery.

Matrix	Type of sample	Spiking level (ng/g)	Found (ng/g)	Trueness (% Bias)	RSD ^a^ (%)	Recovery ^b^ (%)	Number of samples ^c^
Pasta	pooled	1.0	0.98	−2.4	5.6		18
	pooled	10	10.6	+6.3	2.9		18
	pooled	100	103	+3.5	3.5		18
	individual	5.0	4.92	−1.5	3.3	91	21
Rice	pooled	1.0	0.97	−3.4	6.6		18
	pooled	10	10.5	+4.7	4.2		18
	pooled	100	105	+5.2	3.4		18
	individual	5.0	4.77	−4.5	3.1	93	21

^a^ Relative standard deviation; ^b^ Absolute extraction recovery, obtained by comparing results for samples spiked before and after extraction, respectively; ^c^ Six or seven replicates on three different days.

### 2.4. LOD and LOQ

The limit of detection (LOD) for the method was experimentally determined by spiking blank rice and pasta with cereulide standard at 0.5, 0.1, 0.05 and 0.01 ng/g, and was found to be 0.1 ng/g. At this concentration level of cereulide all three confirmation fragments were visible and the fragment ion area quotients of the quantification fragments and of the two confirmation fragments were within the interval ±20% (see Table 2 for details) and with a S/N of ≥20 (calculated from the measurement of the peak-to-peak noise around the retention time of the analyte). Although the cereulide was not quantified on this level the same criteria for the peak identification was applied in order to retain the specificity of the detection. The limit of quantification (LOQ) for the method was set to 1 ng/g, defined as the lowest level at which acceptable precision and trueness was experimentally proven.

**Table 2:** The summary of the *m*/*z* values and parameter settings in the MS and MS/MS analysis. The product ions used as quantification fragments are indicated by bold figures.

Analyte	Precursor ion (*m*/*z*)	Product ion (*m*/*z*)	Cone (V)	Collision (eV)
Cereulide	1170.7	357.3	64	68
	1170.7	314.2	64	72
	1170.7	**172.15**	64	80
Cereulide-C13	1176.7	315.2	56	70
	1176.7	**172.15**	56	80
	1176.7	72.1	56	80

### 2.5. Specificity

The specificity of the method was investigated using 21 blank food samples from each type of matrix that were analyzed regarding the presence of fragments associated with the cereulide toxin or the internal standard. The samples were from authentic cooked pasta or rice meals prepared in individual households or restaurants. In most cases they contained smaller amounts of other meal components, e.g., meat and tomato sauce. No interferences were found at the concentration levels ≥LOD in any of these samples. Based on these results, the method is considered as specific for cereulide toxin.

### 2.6. Incurred Samples

Finally, the method was tested under conditions similar to real food contamination by analysis of cereulide in blank food samples incubated with *B. cereus*. The food samples had been inoculated with cultures of three different strains of *B. cereus* and incubated for 48 h at 25 °C and 30 °C, where after the sample extracts were prepared and analyzed as described in Sample preparation and UPLC-MS/MS. Whereas there was no evidence of the cereulide presence in two of the incubated samples (SLV517 and SLV516), the third sample (CCUG52702) contained high amount of cereulide (beyond the highest validated concentration level of the method). This demonstrates the rapidness of the cereulide production process to concentrations far above the illness-inducing doses for humans that easily can be reached in very small amounts of foods when stored at ambient temperature. The two of the *B. cereus* inoculated samples in which the cereulide toxin was not found (SLV517 and SLV516) were presumably containing non-cereulide producing *B. cereus* strains that, on the other hand, might possess the ability to produce enterotoxins (diarrheal toxins), as these strains were isolated from samples suspected to cause the foodborne illness. However, this type of toxins cannot be identified with the present method.

## 3. Experimental Section

### 3.1. Chemicals and Materials

The synthetic cereulide peptide standard and the ^13^C_6_-labeled internal standard were purchased from Chiralix B. V. (Nijmegen, The Netherlands). Valinomycin was purchased from Sigma-Aldrich (Stockholm, Sweden). Acetonitrile was of LC-MS grade purchased from Fischer Scientific (Loughborough, Leicester, UK), and all other chemicals were of pro-analysis grade and obtained from Merck (Darmstadt, Germany). Water was purified with Milli-Q purification system (Millipore, Solna, Sweden). Stock solutions were prepared by dissolving cereulide standard in methanol. The solutions were stored at −20 °C until analysis when they were further diluted to prepare solutions at a concentration range of 0–500 ng/g.

### 3.2. Validation Samples

Pool samples of rice and pasta, respectively, were prepared by cooking raw products purchased at a local store according to the instructions on the packages, pooling the three different brands and homogenizing each pool in a mixer.

*Individual samples* of authentic food were donated from lunch boxes prepared by different persons. In total, 21 samples of rice and as many of pasta were collected. The samples contained mainly pasta or rice but also smaller amounts of sauce, fat and protein were included which was regarded as a robustness challenge for the method.

Spiked samples were prepared by adding cereulide dissolved in methanol to homogenized food samples. After mixing, the spiked sample was left at room temperature for 30 min for equilibration before extraction and analysis as described in the next sections. For determination of extraction recovery spiking was also done in blank sample extracts.

In order to test the usefulness of the method for the analysis of real samples contaminated with cereulide toxin producing strains of *Bacillus cereus* three different *B. cereus* strains were grown on blood agar plates (Oxoid, Basingstoke, UK). Two–three pure colonies of each of the bacteria strains, previously stored at −70 °C, were transferred and suspended in 10 mL of saline buffer (0.9% NaCl). From each of these suspensions 500 μL were used to contaminate 3 g portions of rice or pasta, respectively, and the samples were incubated in 25 °C and 30 °C during 48 h. Thereafter, the internal standard was added and the extraction procedure was applied for these samples and positive/negative control samples following the analysis and evaluation as described below.

### 3.3. Sample Extraction

Homogenized food samples (3 g) were placed in a sample tube and ^13^C_6_-Cereulide internal standard solution was added to a concentration of 10 ng/g (50 μL of a 0.6 μg/mL methanol solution). The extraction procedure was thereafter started by adding 15 mL of methanol and mixing samples for 30 s using a vortex mixer. The samples were then shaken for 15 min and centrifuged at 4000 × *g* for 15 min. Finally, 500 μL sample extract was mixed with 500 μL of water before injection in the UPLC-MS/MS system.

### 3.4. UPLC-ESI-MS/MS

Analysis was performed using an Acquity UPLC BEH C8 1.7 μm, 2.1 × 50 mm column and Waters UPLC I-Class (Waters, Milford, MA, USA) with Waters Xevo TQ-S mass spectrometer system (Waters) operating in ESI+ mode. The ionization parameters were set to: capillary voltage 3 kV, desolvation temperature 450 °C, desolvation gas flow rate 800 L/h, source temperature of 150 °C, cone gas flow rate 150 L/h, collision gas flow rate 0.15 mL/min and the collision gas pressure 3.5 ×10^−3^ mBar. The column temperature was maintained at 40 °C and the injection volume was 10 μL. The analysis was performed in multiple-reaction-monitoring mode and argon was used as the collision gas. Mobile phase gradient consisted of 1 mM ammonium formate in water containing 0.05% formic acid (A) and 0.05% formic acid in acetonitrile (B). The flow rate was set to 0.5 mL/min. The gradient started at 50% B for 1 min, linearly increased to 95% B over 3 min and kept at 95% B for 2 min and then reduced to 50% B over 0.1 min, following an equilibration period of 1 min. The highest abundant ions in MS1 were the ammonium adducts [M+NH_4_^+^] of the cereulide and the ^13^C_6_-cereulide internal standard corresponding to *m*/*z* 1170.7 and 1176.7 respectively, which were consequently selected as precursor ions. For identification and quantification in MS2 the product ions *m*/*z* 1170.7→172.15 and 1176.7→172.15 were selected. Table 2 summarizes the details of the MS and MS/MS analysis. For quantitative analysis Targetlynx v 4.1 software (Waters, 2011) was applied. Solutions of cereulide (0, 1, 5, 20, 100 and 500 ng/g) in 50% methanol, all containing ^13^C_6_-cereulide at 10 ng/g, were injected to obtain calibration curves. These were constructed by plotting peak area ratios of cereulide to internal standard against concentration ratios of the analyte to the internal standard using linear regression.

For the confirmation of the analyte findings the quotients of areas of the quantification fragments and the two confirmation fragments were used which should not differ more than ±20% of the average for the quotients of the other calibration points in the curve. Additionally, the signal-to-noise ratio (S/N) has to be ≥20 in the positive control sample for all the fragments.

## 4. Conclusions

An in-house validation of a fast and straightforward UPLC-ESI-MS/MS method for qualitative and quantitative determination of cereulide toxin from *Bacillus cereus* is presented. The method is validated for food matrices based on rice and pasta, which stand for the vast majority of the food poisonings involving cereulide in the world. A, nowadays, commercially available synthetic cereulide standard and ^13^C_6_-labeled internal standard have been used being the ideal standards for quantitative MS analysis of cereulide toxin. Although, the robustness of the method is not evaluated in this study it is strengthened by use of the ^13^C_6_-cereulide internal standard, which enables revealing of possible robustness related method deviations. This involves the advantages of minimizing the risk of false negative results as well as it equalizes the prerequisites for optimal electrospray ionization and MS-detection of cereulide. The presented method is time and cost effective and easy to perform which together with its high sensitivity, specificity and precision makes it convenient to apply in emergent situations where it is important to reveal or reject the presence of the cereulide as the suspected causative agent in food poisonings in order to protect human health.

## References

[1] Kramer J.M., Gilbert R.J., Doyle M.P. (1989). *Bacillus cereus* and other Bacillus species. Foodborne Bacterial Pathogens.

[2] Stenfors Arnesen L.P., Fagerlund A., Granum P.E. (2008). From soil to gut: *Bacillus cereus* and its food poisoning toxins. FEMS Microbiol. Rev..

[3] (2005). Opinion of the Scientific Panel on Biological Hazards on *Bacillus cereus* and other *Bacillus* spp. in foodstaffs. EFSA J..

[4] Dierick K., van Coillie E., Swiecicka I., Meyfroidt G., Devlieger H., Meulemans A., Hoedemaekers G., Fourie L., Heyndrickx M., Mahillon J. (2005). Fatal family outbreak of *Bacillus cereus*-associated food poisoning. J. Clin. Microbiol..

[5] Mahler H., Pasi A., Kramer J.M., Schulte P., Scoging A.C., Bar W., Krahenbuhl S. (1997). Fulminant liver failure in association with the emetic toxin of *Bacillus cereus*. N. Engl. J. Med..

[6] Shiota M., Saitou K., Mizumoto H., Matsusaka M., Agata N., Nakayama M., Kage M., Tatsumi S., Okamoto A., Yamaguchi S. (2010). Rapid detoxification of cereulide in *Bacillus cereus* food poisoning. Pediatrics.

[7] Granum P.E., Lund T. (1997). *Bacillus cereus* and its food poisoning toxins. FEMS Microbiol. Lett..

[8] Jääskeläinen E.L., Häggblom M.M., Andersson M.A., Salkinoja-Salonen M.S. (2004). Atmospheric oxygen and other conditions affecting the production of cereulide by *Bacillus cereus* in food. Int. J. Food Microbiol..

[9] Agata N., Mori M., Ohta M., Suwan S., Ohtani I., Isobe M. (1994). A novel dodecadepsipeptide, cereulide, isolated from *Bacillus cereus* causes vacuole formation in HEp-2 cells. FEMS Microbiol. Lett..

[10] Agata N., Ohta M., Mori M., Isobe M. (1995). A novel dodecadepsipeptide, cereulide, is an emetic toxin of *Bacillus cereus*. FEMS Microbiol. Lett..

[11] Ehling-Schulz M., Fricker M., Scherer S. (2004). *Bacillus cereus*, the causative agent of an emetic type of food-borne illness. Mol. Nutr. Food Res..

[12] Rajkovic A. (2014). Microbial toxins and low level of foodborne exposure. Trends Food Sci. Technol..

[13] Andersson M.A., Hakulinen P., Honkalampi-Hämäläinen U., Hoornstra D., Lhuguenot J.-C., Mäki-Paakkanen J., Savolainen M., Severin I., Stammati A.-L., Turco L. (2007). Toxicological profile of cereulide, the *Bacillus cereus* emetic toxin, in functional assays with human, animal and bacterial cells. Toxicon.

[14] Jääskeläinen E.L., Teplova V., Andersson M.A., Andersson L.C., Tammela P., Andersson M.C., Pirhonen T.I., Saris N.E.L., Vuorela P., Salkinoja-Salonen M.S. (2003). *In vitro* assay for human toxicity of cereulide, the emetic mitochondrial toxin produced by food poisoning *Bacillus cereus*. Toxicol. in Vitro.

[15] Paananen A., Mikkola R., Sareneva T., Matikainen S., Hess M., Andersson M., Julkunen I., Salkinoja-Salonen M.S., Timonen T. (2002). Inhibition of human natural killer cell activity by cereulide, an emetic toxin from *Bacillus cereus*. Clin. Exp. Immunol..

[16] Delbrassinne L., Andjelkovic M., Rajkovic A., Dubois P., Nguessan E., Mahillon J., van Loco J. (2012). Determination of *Bacillus cereus* emetic toxin in food products by means of LC–MS^2^. Food Anal. Methods.

[17] Naranjo M., Denayer S., Botteldoorn N., Delbrassinne L., Veys J., Waegenaere J., Sirtaine N., Driesen R.B., Sipido K.R., Mahillon J. (2011). Sudden death of a young adult associated with *Bacillus cereus* food poisoning. J. Clin. Microbiol..

[18] Andersson M.A., Mikkola R., Helin J., Andersson M.C., Salkinoja-Salonen M. (1998). A novel sensitive bioassay for detection of *Bacillus cereus* emetic toxin and related depsipeptide ionophores. Appl. Environ. Microbiol..

[19] Kawamura-Sato K., Hirama Y., Agata N., Ito H., Torii K., Takeno A., Hasegawa T., Shimomura Y., Ohta M. (2005). Quantitative analysis of cereulide, an emetic toxin of *Bacillus cereus*, by using rat liver mitochondria. Microbiol. Immunol..

[20] Hughes S., Bartholomew B., Hardy J.C., Kramer J.M. (1988). Potential application of a HEp-2 cell assay in the investigation of *Bacillus cereus* emetic-syndrome food poisoning. FEMS Microbiol. Lett..

[21] Beattie S.H., Williams A.G. (1999). Detection of toxigenic strains of *Bacillus cereus* and other Bacillus spp. with an improved cytotoxicity assay. Lett. Appl. Microbiol..

[22] Bauer T., Stark T., Hofmann T., Ehling-Schulz M. (2010). Development of a stable isotope dilution analysis for the quantification of the *Bacillus cereus* toxin cereulide in foods. J. Agric. Food Chem..

[23] Haggblom M.M., Apetroaie C., Andersson M.A., Salkinoja-Salonen M.S. (2002). Quantitative analysis of cereulide, the emetic toxin of *Bacillus cereus*, produced under various conditions. Appl. Environ. Microbiol..

[24] Shaheen R., Andersson M.A., Apetroaie C., Schulz A., Ehling-Schulz M., Ollilainen V.M., Salkinoja-Salonen M.S. (2006). Potential of selected infant food formulas for production of *Bacillus cereus* emetic toxin, cereulide. Int. J. Food Microbiol..

[25] Biesta-Peters E.G., Reij M.W., Blaauw R.H., In ’t Veld P.H., Rajkovic A., Ehling-Schulz M., Abee T. (2010). Quantification of the emetic toxin cereulide in food products by liquid chromatography-mass spectrometry using synthetic cereulide as a standard. Appl. Environ. Microbiol..

[26] Zuberovic A. (2009). Surface Modified Capillaries in Capillary Electrophoresis Coupled to Mass Spectrometry: Method Development and Exploration of the Potential of Capillary Electrophoresis as a Proteomic Tool. Doctoral Thesis.

